# Intelligence quotient scores among early-treated phenylketonuria patients: results from a systematic literature review

**DOI:** 10.1186/s13023-025-03830-0

**Published:** 2025-06-20

**Authors:** Fiona O’Sullivan, Ioannis Tomazos, Francjan J. van Spronsen, Shelagh M. Szabo, Maanasa Venkataraman, Lavanya Huria, Neil Smith, Lachlan Molony, Kim Ingalls, Kathleen Somera-Molina, Rongrong Zhang, Cary O. Harding

**Affiliations:** 1 Broadstreet HEOR, Vancouver, BC Canada; 2 PTC Therapeutics Inc, Warren, NJ USA; 3 Beatrix Children’s Hospital, University Medical Centre Groningen, University of Groningen, Groningen, Netherlands; 4 PTC Therapeutics Sweden AB, Askim, Sweden; 5 Department of Molecular and Medical Genetics and Department of Pediatrics, Oregon Health & Science University, Portland, OR USA

**Keywords:** Phenylketonuria, PKU, IQ, Diet, Adherence

## Abstract

**Background:**

Phenylketonuria (PKU) is a rare condition that causes the accumulation of phenylalanine; without prompt diagnosis and treatment following birth, severe neurologic and cognitive impairments occur. While dietary management can help reduce Phe levels, adherence is challenging and deficits in cognitive function often remain. The importance of the exact features of dietary management, treatment, and control at different time points with respect to eventual IQ scores has not been established. The objective of the present study was to review and describe published data on the impact of PKU on cognition as measured by IQ among PKU patients receiving early dietary management.

**Methods:**

A systematic literature review was conducted following PRISMA guidelines to examine IQ among patients with PKU. Instruments used to assess IQ included the Wechsler Intelligence Scale, Culture Fair Intelligence Test and Stanford Binet Test. Results were reported overall and by subgroups.

**Results:**

Twenty-five out of 28 identified studies could be included in the review. Lower IQ scores were generally observed among those with high phenylalanine levels, although variations in the study populations hinder the ability to make comparisons. Mean IQ scores among those with PKU were consistently lower compared to control groups. Even though all patients in this review received early treatment, those with poor dietary adherence and higher phenylalanine levels tended to show poorer cognitive ability.

**Conclusions:**

Cognition is affected in PKU, despite early and continuous dietary management. Treatments are needed that reduce phenylalanine levels so that the burden of neurocognitive impairment in PKU can be alleviated.

**Supplementary Information:**

The online version contains supplementary material available at 10.1186/s13023-025-03830-0.

## Introduction

Phenylketonuria (PKU) results from an inborn error of phenylalanine (Phe) metabolism, which is caused by two variants in the phenylalanine hydroxylase *PAH*- gene. This ultimately results in a deficiency of PAH and consequently, the accumulation of Phe occurs. If left untreated, most patients with PKU will develop severe neurologic and cognitive impairments, social and executive functioning deficits, as well as behavioral and psychiatric issues [1–3]. PKU is also associated with movement issues, dermatologic conditions, and seizures [4].

While the mechanisms underlying the neurologic and cognitive impairments experienced by individuals with PKU are not yet completely understood, inhibition of the entry of certain amino acids across the blood brain barrier, reduction in the synthesis of myelin and other brain proteins, Phe toxicity, reduction of glutamatergic synapsis transmission, oxidative stress, and pyruvate kinase inhibition and calcium homeostasis dysregulation are thought to be factors [5]. As evidence has shown that every four-week delay in commencing treatment can cause a decline in IQ score of approximately 4 points [6], most patients with PKU are diagnosed at birth and begin dietary management or pharmaceutical treatment within a week to 10 days of birth per current guideline recommendations [7–13]. Early intervention aims to prevent the negative impacts of PKU by attempting to keep Phe levels within target ranges. However, adherence to strict dietary management can be challenging for patients and their caregivers and the burden of maintaining such a diet has been shown to have a negative impact on health-related quality-of-life (HRQoL) [14–16]. A negative trend was found between low Phe concentrations in early years of early treated PKU patients and adult emotional and social outcomes [17].

Guidelines for the management of PKU differ between countries and jurisdictions. In the United States, guidelines recommend a target blood Phe concentration of 120 − 360 μmol/L for patients of any age [11]. Similarly, European guidelines recommend pregnant individuals and those < 12 years of age to target a blood Phe concentration of 120 − 360 μmol/L. For those > 12 years old, the European recommendations are less strict, with an upper target level of 600 μmol/L [13]. However, questions remain surrounding the impact of the higher target level of Phe on clinical outcomes, and of stricter dietary management on patient HRQoL, and consensus on the identification of an appropriate upper target blood Phe concentration level for all age groups has yet to be achieved [18–21]. There is also some debate among researchers about the overall benefit of a low Phe diet and protein substitutes to patients, regardless of age [22]. Additionally, studies have shown wide variability in blood Phe levels required for treatment initiation as well as target levels of blood Phe across clinics, both in Europe and the United States (US) [23, 24].

As previously noted, cognitive impairment represents one of the negative impacts of PKU; an inverse correlation between blood and plasma Phe levels and IQ has been established [6, 25, 26]. Higher rates of intellectual disability and significantly lower IQ scores have also been observed in patients with PKU versus control groups [1]. In particular, deficits in working memory, reasoning/planning, attention, and processing speed have been observed in children with PKU [27, 28] and in vigilance, working memory, and motor skills in adults [29].

With early dietary intervention and treatment, the severe cognitive impairments that may occur with untreated PKU are preventable [1]. However, deficits in cognitive function of varying magnitude have still been reported among those who are treated [30]. Likewise, executive function and social functional deficits, may also remain [31]. Indeed, studies have found that even early-treated PKU patients on diet therapy with good metabolic control are outperformed by healthy controls in measures of cognitive ability [30, 32]. Furthermore, patients who are diagnosed and treated early tend to have IQ scores within the average range for the general population; nonetheless these are often lower than scores from healthy controls such as peers and family members. This suggests the existence of an altered cognitive phenotype, even with early intervention [6, 33–35].

The precise molecular pathophysiological mechanisms resulting in cognitive impairment despite early treatment in some patients are not fully understood [36]. As cognitive ability is related to the control of Phe levels in early childhood, adherence to dietary management in early life and through adulthood is crucial to protect functioning, although strict dietary adherence remains a challenge for many patients with PKU [14, 15, 30]. However, the impact of variations in aspects of dietary management or treatment at different time points with respect to eventual IQ scores has not been synthesized. In addition, recent data on the cognitive abilities of those with PKU who underwent early dietary management or treatment, compared to those without PKU, have not been described in detail. The objective of this review was to synthesize published data on the impact of PKU on cognition as measured by IQ among PKU patients receiving early dietary management.

## Methods

### Search and study identification

A systematic literature review (SLR) was conducted following PRISMA guidelines to examine IQ among patients with PKU. Database-specific search strategies were developed and implemented on March 2, 2023, for a review of the following databases via Ovid**: 1.** Medical Literature Analysis and Retrieval System Online, MEDLINE®, **2.** Excerpta Medica, Embase, **3.** Northern Light Life Sciences Conference Abstracts. A search in MEDLINE in-process was also conducted to ensure that the most recent data were captured (Tables S1 and S2). Non-English publications were excluded and a year restriction of 1986 onwards was included to align with the discovery of the first mutation in the *PAH* gene [37, 38]. Abstracts from the following conferences from 2021 to 2023 were examined in detail were: Society for Inherited Metabolic Disorders (SIMD), Society for the Study of Inborn Errors of Metabolism (SSIEM), International Congress of Inborn Errors of Metabolism (ICIEM) and the Annual Clinical Genetics Meeting (ACMG)***.*** Pre-tested study design filters developed and published by the Scottish Intercollegiate Guidelines Network (SIGN) were used [39].

The search strategy was reviewed and tested by a second researcher not involved in its development and reviewed by the study team prior to its implementation. De-duplication was carried out using the algorithm established by Bramer et al., as recommended by EUnetHTA guidelines [40, 41]. Identified studies from the search were evaluated against the inclusion criteria (Population, Intervention, Comparators, Outcomes, Study design; PICOS) as shown in Table S3. Abstract and full text screening were carried out in duplicate by MV, LH, and EK; a third researcher or study team member (FOS, CH, FVS) provided arbitration when discrepancies occurred. Observational studies assessing IQ among early-diagnosed and early-treated patients (where “early-diagnosed” and “early-treated” are the terms used in the study or where diagnosis and treatment began within 3 months of birth) with PKU (blood Phe level > 600 micromole per liter (μmol/L) at screening) that measured IQ and fit the PICOS criteria were included. Double data extraction (one primary extractor and a second quality check reviewer) into a customized Microsoft® Excel® data extraction workbook was performed for all data source, demographic, clinical, and outcomes data of interest from the eligible studies.

### IQ scales

The Wechsler Intelligence Scale, Culture Fair Intelligence Test and Stanford Binet Test are the most frequently-reported measures across studies reporting on IQ in PKU; and were therefore specified as the scales of interest for the present review. For each of these scales, IQ scores are standardized such that a score of 100 represents ‘average intelligence.’ A standard deviation (SD) of 15 is used for the Wechsler scale, meaning that 68% of scores fall within one SD of the mean (e.g., between 85 and 115). An SD of 16 is used for both the Culture Fair Intelligence Test and Stanford Binet Test. The Wechsler Intelligence Scale comprises Verbal IQ and Performance IQ scales; the Verbal scale measures acquired knowledge, verbal reasoning, and attention to verbal materials, while the Performance scale measures fluid reasoning, spatial processing, attentiveness to details, and visual-motor integration. Both the Verbal and Performance IQ scales combine to form the Full IQ scale score. Versions of the Wechsler Intelligence Scale [42, 43] differ according to age group and include the Wechsler Intelligence Scale for Children (WISC), the Wechsler Preschool and Primary Scale of Intelligence (WPPSI), and the Wechsler Adult Intelligence Scale (WAIS). The Culture Fair Intelligence Tests consist of three scales (Scale I-III) with non-verbal visual puzzles. Scale I includes items such as mazes, copying symbols and other non-verbal tasks. Scales II and III include a classification of drawings, and carrying out sequences of drawings and patterns. The Stanford Binet Test measures five weighted factors including knowledge, quantitative reasoning, visual-spatial processing, working memory, and fluid reasoning.

### Data comparison

Mean IQ scores derived from the included studies were examined. Results were reported by study overall, age group and subgroups (e.g. among children vs. adults), depending on how the data were reported in the original articles. All studies included are included in the results section, however, if presented data within articles was incomparable to other included studies, these results were only descriptively described. The following terms were used to describe how the reported data were grouped: ‘All studies,’ where data was included from all studies describing IQ measures for all groups and subgroups, within a given age group; ‘Overall population,’ where data was included from studies that only reported IQ measures for the overall PKU population within a given age group; and, ‘Restricted subgroups,’ where data was included from studies that restricted their cohorts to particular subgroups such as patients who went on or off their diet, or according to high/low Phe levels within a given age group. The percentage of studies where patients had mean IQ scores < 100 (selected as the threshold as the standardized mean of 100 is defined as ‘average intelligence’) was examined. Differences in IQ between PKU and control groups (the mean IQ scores siblings or healthy controls, rather than standardized general population measures) from the same studies were explored.

## Results

### Study inclusion

**Fig. 1:**
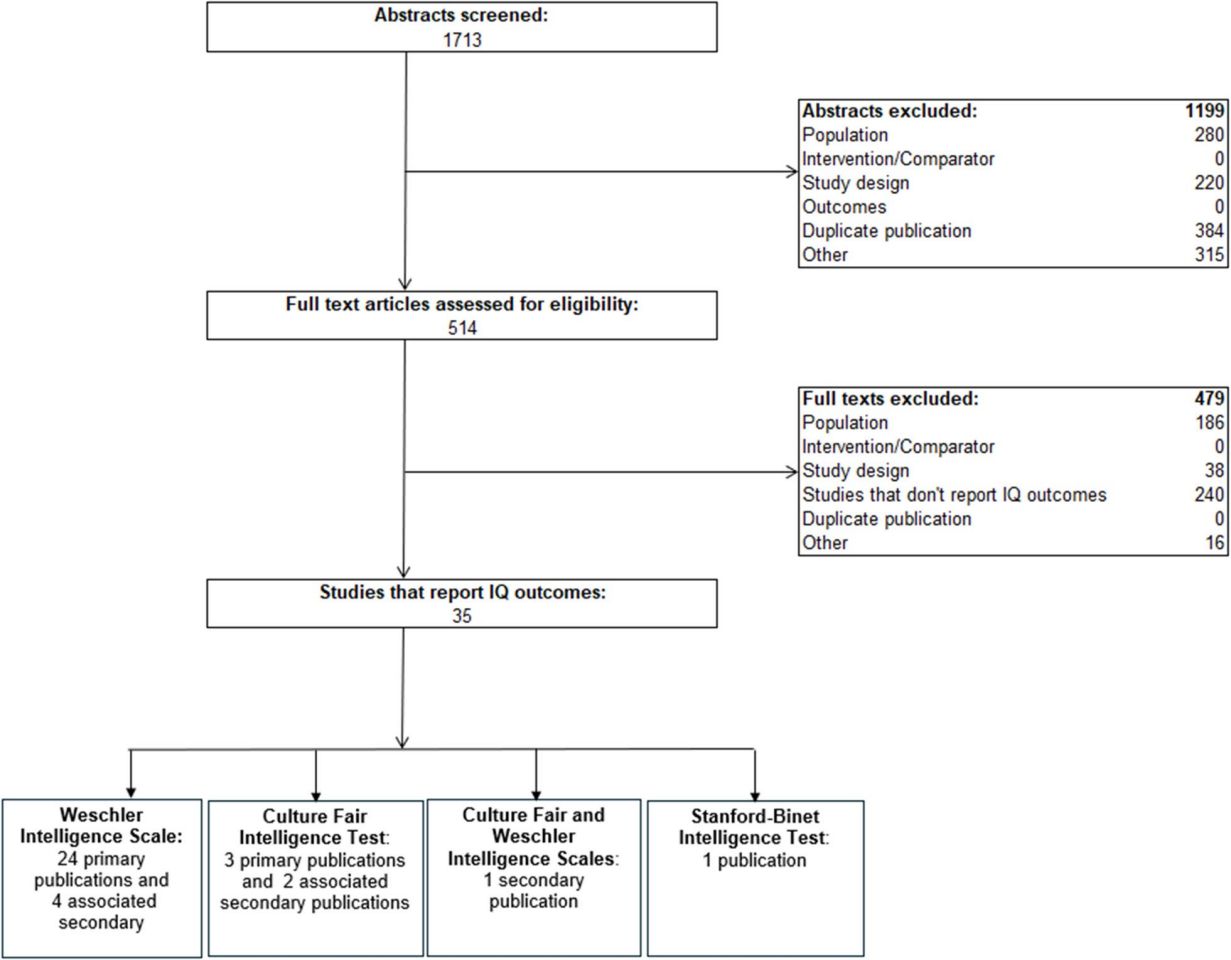
PRISMA diagram of study selection. *35 publications were included in the SLR. However, 3 studies were described descriptively below and not included in the analysis figures as the data that was presented in those articles was incomparable to other included studies

Twenty-eight studies from 35 publications reported IQ outcomes (Fig. 1) [34, 35, 44–75]. Twenty-four studies from 28 publications reported IQ scores on the Wechsler Intelligence Scale [34, 35, 44–69], 3 studies (and 2 associated publications) reported data on the Culture Fair Intelligence Test [51, 71–74], 1 study reported on the Stanford Binet Intelligence Test [70], and 1 study presented data on both the Culture Fair Intelligence Test and the Wechsler Intelligence Scale [75]. With the exception of 1 study, [69] all patients in the included studies reported dietary management and no other treatments; van Vliet et al. 2022 reported on 15/31 patients being treated with tetrahydrobiopterin. In that study, only subscales of the Weschler Intelligence scale were reported. [69]. Of the 28 primary studies, 10 were cross-sectional [34, 35, 45, 53, 56, 59–62, 69, 76], 6 were retrospective [44, 46, 48, 52, 63, 64, 67, 71, 73, 75, 77] and 1 was prospective [65, 66, 70]. Eleven followed patients over time or collected data at different ages for the same cohort; among these, 8 studies were retrospective [49, 57], and 3 studies were prospective [55, 74].

Fourteen studies were single-center [35, 44, 46, 49, 52, 54, 55, 57, 60–63, 66–68, 75] and 3 were multi-center [48, 69, 70]; in 11 studies these details were not reported [34, 44, 47, 50, 51, 53, 56, 58, 59, 64, 65, 71–74, 77]. Sample size ranged from 4 to 103 across the included studies.

Results from 3 of the 28 studies identified in this review were not further explored as the presented data was incomparable to other included studies. Firstly, Saudubray et al*.* examined IQ at different ages (age 5, 7, 9, 11 years); this was not directly comparable data to other studies included in this review, although it was noted that IQ scores remained relatively consistent across different ages [48]. Pardo Campos et al*.* examined the Comprehension subtest of the Wechsler Intelligence Scale among PKU patients and controls; only *p*-values for comparisons were reported, and the comparisons between groups were non-significant [76]. Finally, Van Vliet et al*.* reported the Block Design and Vocabulary subtests of the Wechsler Intelligence Scale; no significant differences were present between healthy controls and PKU patients [69].They also reported finding no differences in IQ between those treated with BH4 and those not treated.

### IQ in children with PKU

#### WISC full scale IQ overall

**Fig. 2:**
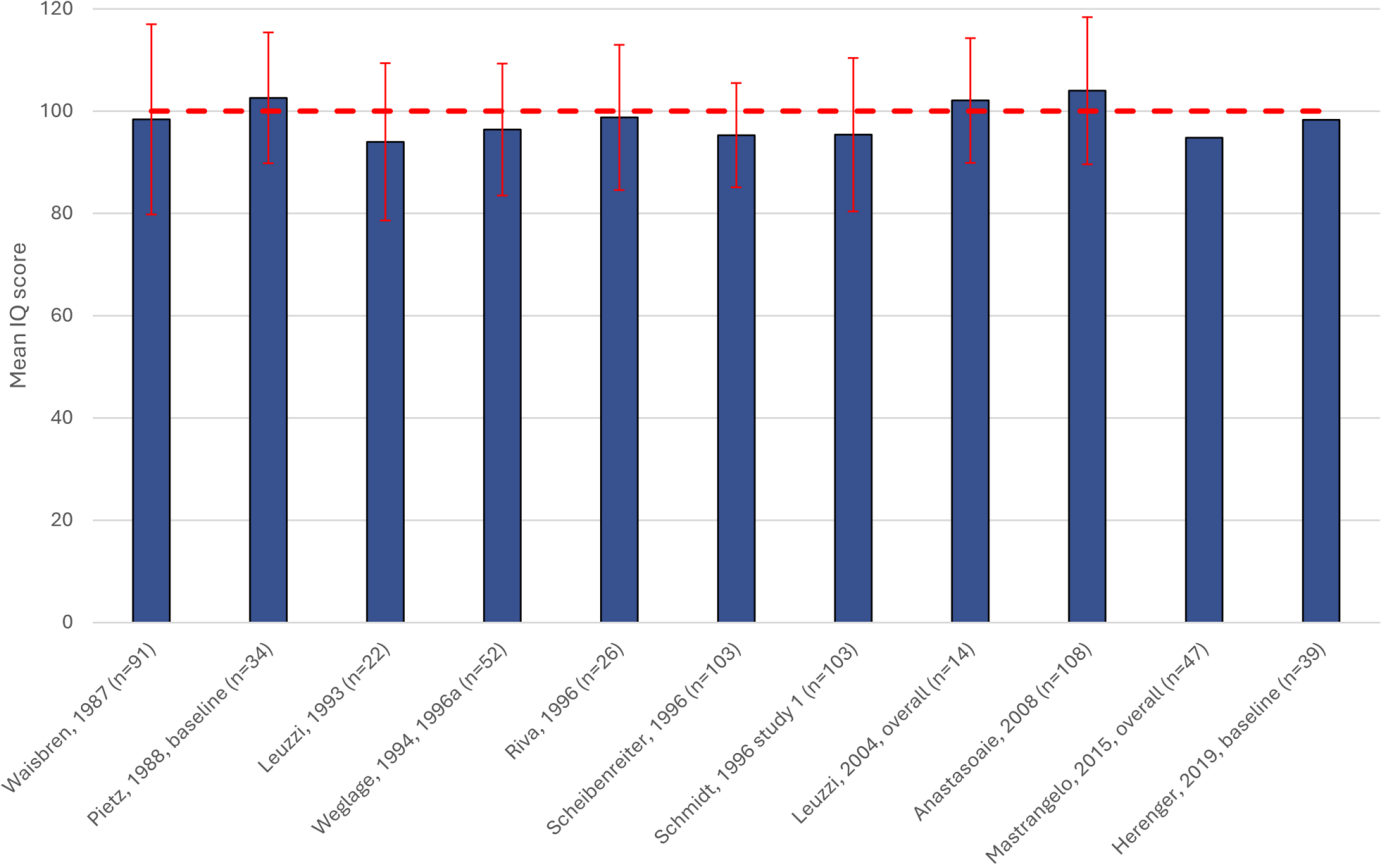
Mean (SD) Wechsler Intelligence full scale IQ in overall populations of children with PKU. Dotted line (----) indicates the standardized ‘average intelligence’ score [93]

Eleven studies reported IQ measures in overall samples of children with PKU (Fig. 2), with mean (SD) IQ scores ranging from 94.0 (18.6) in a cross-sectional study in children aged 5–16 years to 104.0 (15) from a single-center study in children aged 3–16 years old. Eight out of 11 studies (72.7%) reported mean IQ < 100 in children (Fig. 2) [34, 45, 46, 49, 52, 56, 57, 63–65, 67].

#### WISC full scale IQ stratified by dietary control and diet and Phe levels

Two studies reported IQ among subgroups of children with PKU defined by dietary control (Figure S1). In one study, mean IQ scores were similar for those with good vs poor diet control [75]; in the second study, a difference in IQ scores was observed among the different dietary control subgroups [65]. Two studies reported IQ scores among early-treated children with optimal and suboptimal Phe levels (Figure S2) defined as a mean index of dietary control (IDC) of 4.7 mg per deciliter (mg/dl) and an IDC of 9.0 mg/dl, respectively, by Pietz et al*.* and above and below 360 μmol/L by Huijbregts et al. [46, 54]. In general, lower IQ scores were observed in those with high Phe levels although the varying subgroups and ages reported prevent further direct comparisons. Three studies reported IQ among subgroups of children with PKU defined by dietary adherence (Figure S3) [44, 45, 49]. The mean (SD) IQ scores in those *on diet* ranged from 97.3 (10.3) to 106.5 (18.2) and were higher than the those *off diet*, which ranged from 87.9 (NR) to 95.6 (15.3).

#### Other IQ measures

Three studies by Weglage et al., examined IQ using the Scale 2 subscale of the Culture Fair Intelligence Test in children and reported mostly average scores (~ 100) (Figure S4) [51, 71, 73]. One study (data not shown) examined IQ using the Stanford Binet Test in children with high Phe vs low Phe; those with high Phe had lower IQ scores (high: 90.4, low: 104.2).

Among all IQ measures, SDs around point estimates highlight the considerable variability in scores among children with PKU.

### IQ in adults with PKU

For all studies reporting on adults with PKU, the variability around mean estimates was large given small sample sizes. Standard deviations for adult IQ scores also demonstrated considerable variability. No data on the Culture Fair Intelligence Test or the Stanford Binet Intelligence Test were presented for adults.

#### WAIS full scale IQ overall

**Fig. 3:**
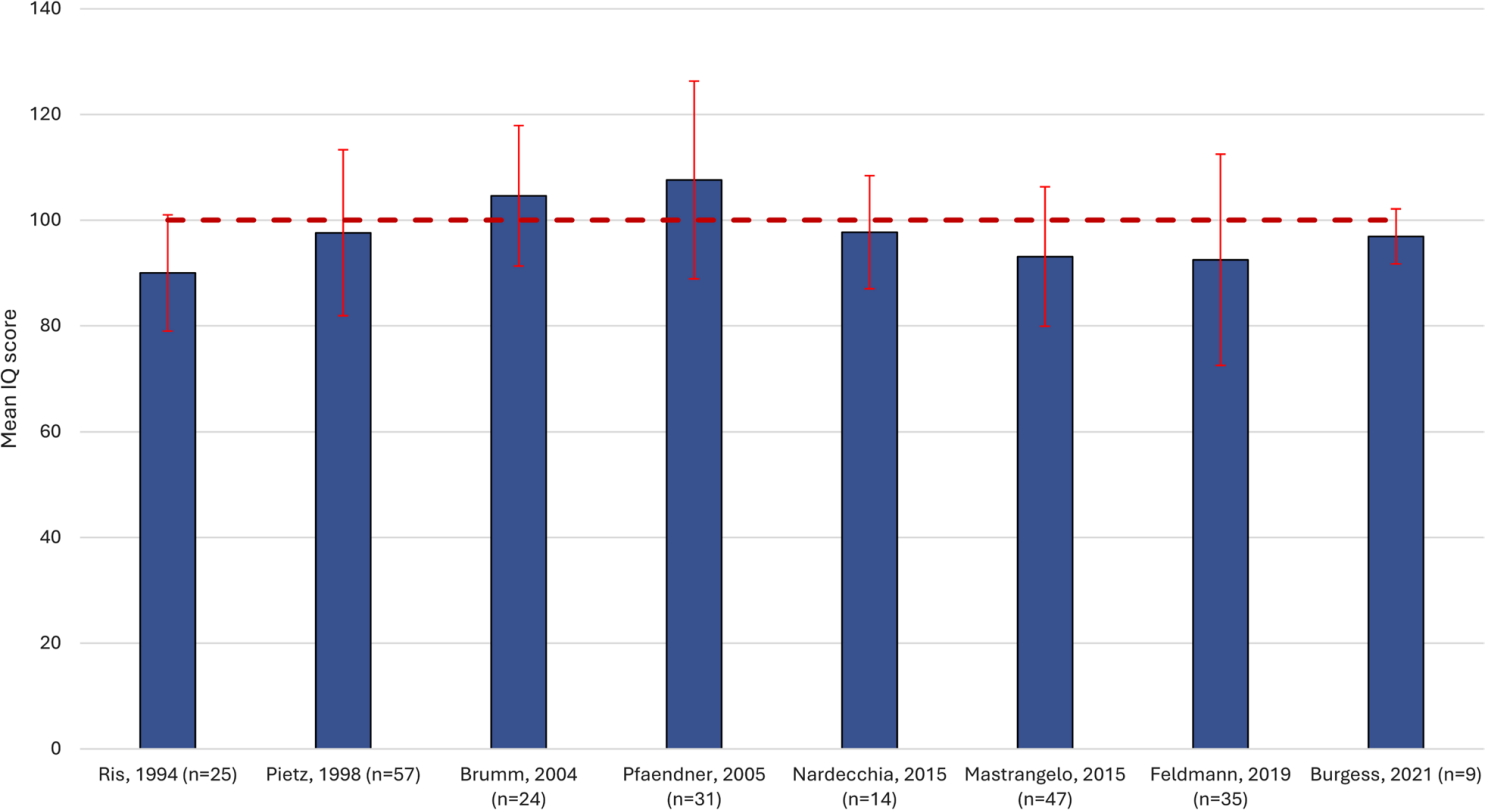
Mean (SD) Wechsler Intelligence full scale IQ in overall populations of adults with PKU. Dotted line (----) indicates the standardized ‘average intelligence’ score [93]

Nine studies reported mean full scale IQ scores in overall samples of adults with PKU who were early-diagnosed and early-treated with dietary management (Fig. 3) [46, 53, 57–59, 61, 62, 66, 77]. The mean (SD) IQ scores ranged from 90.0 (11.0) in a single-center cross-sectional study [62] to 107.6 (18.7), also in a cross-sectional study [59]. Consistent with the findings from pediatric studies, seven of nine studies including adults (77.8%) reported mean IQ scores < 100.

#### WAIS full scale IQ stratified by diet

Among studies reporting results by subgroups of dietary adherence (n = 2), mean (SD) full scale IQ scores were 81.0 (8.0) in a single-center cross-sectional study of patients who terminated their diet early and 112.0 (NR) in a longitudinal retrospective study of adults who were continuously on diet (Figure S5) [47, 62].

### IQ in PKU vs control groups

#### Wechsler intelligence full scale IQ comparisons in children

**Fig. 4:**
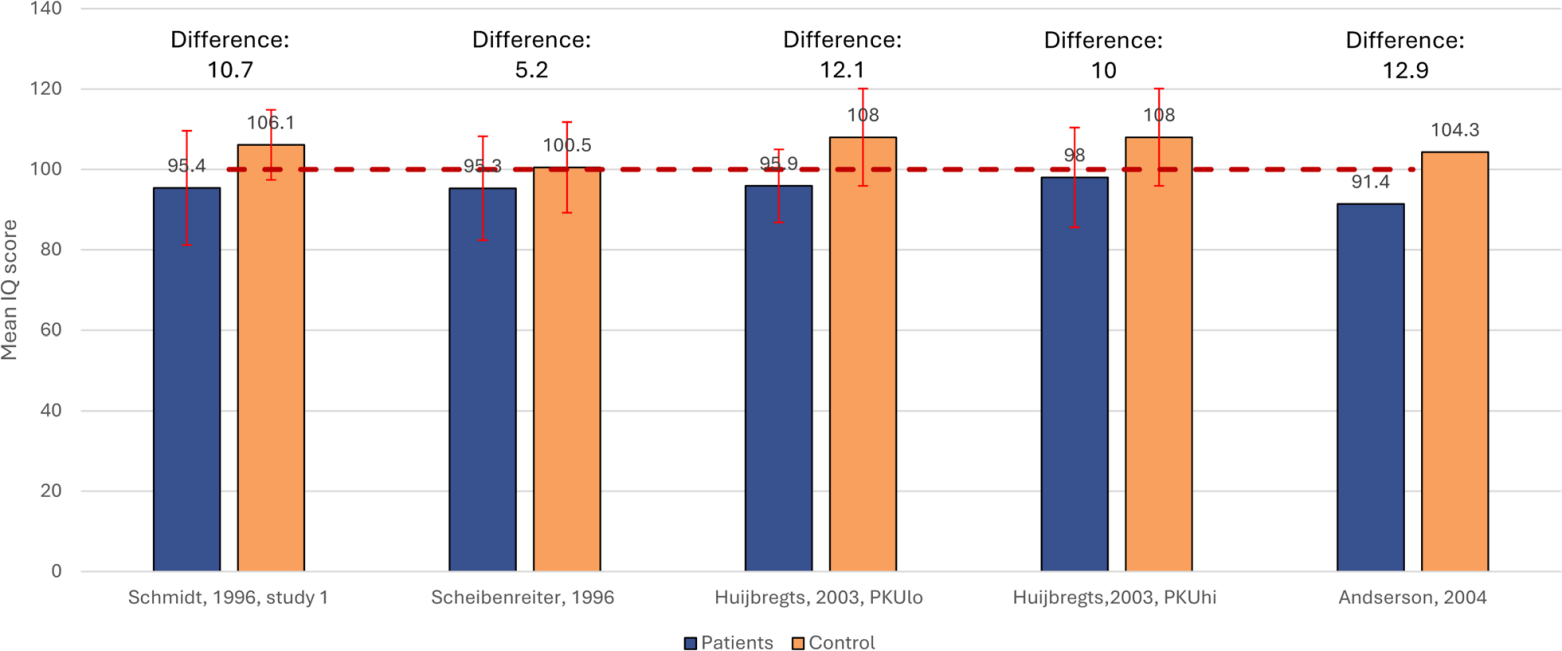
Mean (SD) Wechsler Intelligence Full scale IQ scores in restricted subgroups of children with PKU vs external control groups. Dotted line (----) indicates the standardized ‘average intelligence’ score [93]

Four studies that assessed IQ among children using the Wechsler Intelligence Scale included an external control group (Fig. 4) which consisted of either healthy (age matched or non-age matched) controls or unaffected siblings [34, 35, 55, 64].The difference in mean IQ scores ranged from 5.2 (unaffected siblings) to 12.9 (healthy controls) points higher among control samples vs. among PKU groups.

#### Wechsler intelligence full scale IQ comparisons in adults

**Fig. 5:**
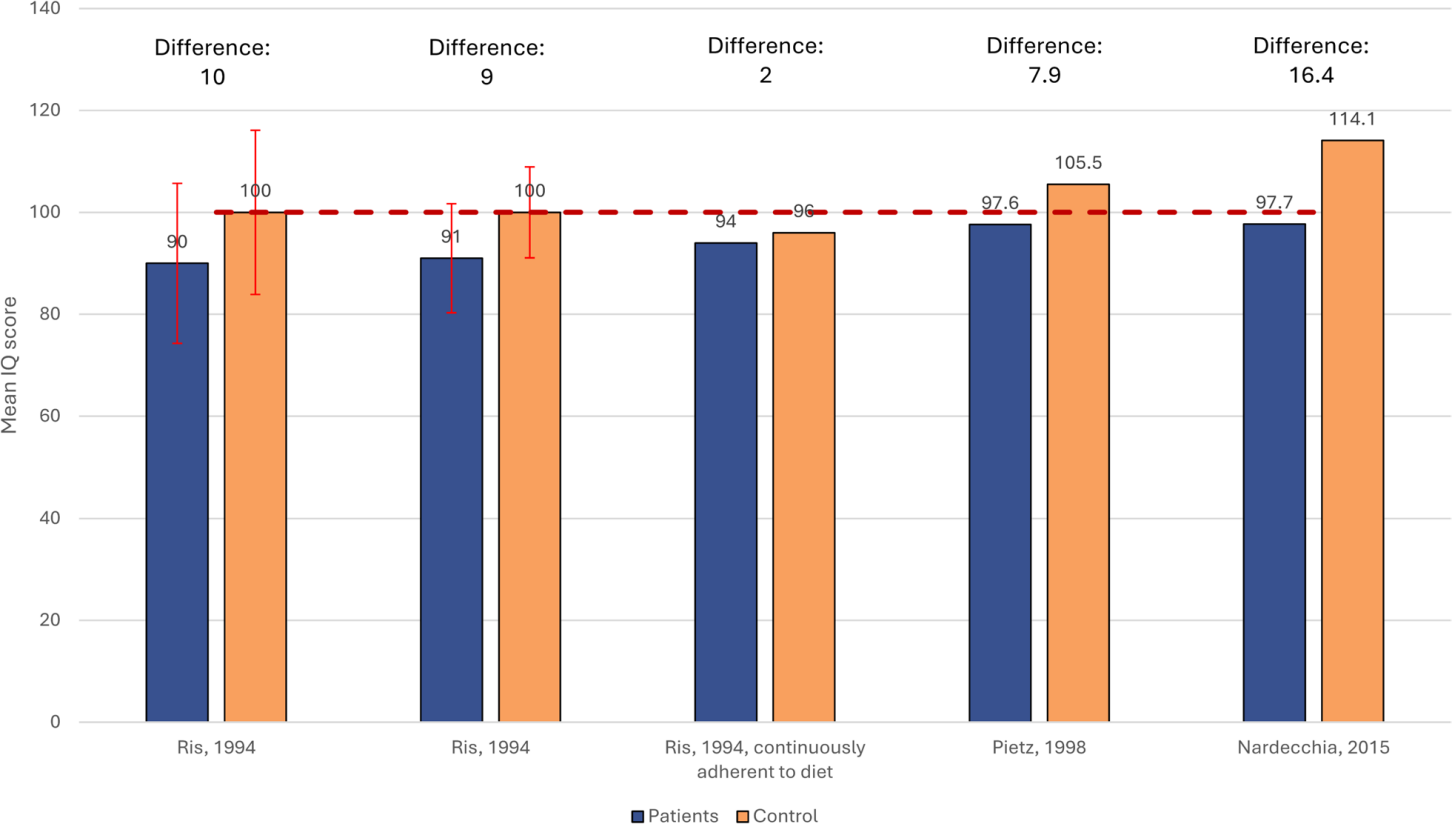
Mean (SD) Wechsler Intelligence Full scale IQ scores in restricted subgroups of adults with PKU vs external control groups. Dotted line (----) indicates the standardized ‘average intelligence’ score [93]

Three studies that assessed IQ among adults using the Wechsler Intelligence Scale included an external control group (Fig. 5) which consisted of either healthy (age and sex matched) controls or unaffected siblings [58, 60, 62]. The mean IQ scores ranged from 2 (unaffected siblings) to 16.4 (age and sex matched healthy controls) higher in controls than among adults with PKU. The differences between PKU and control groups tended to be larger in adults compared to children, with the exception of adults who were continuously diet-adherent.

#### Culture fair intelligence test and stanford binet test comparisons in children

**Fig. 6:**
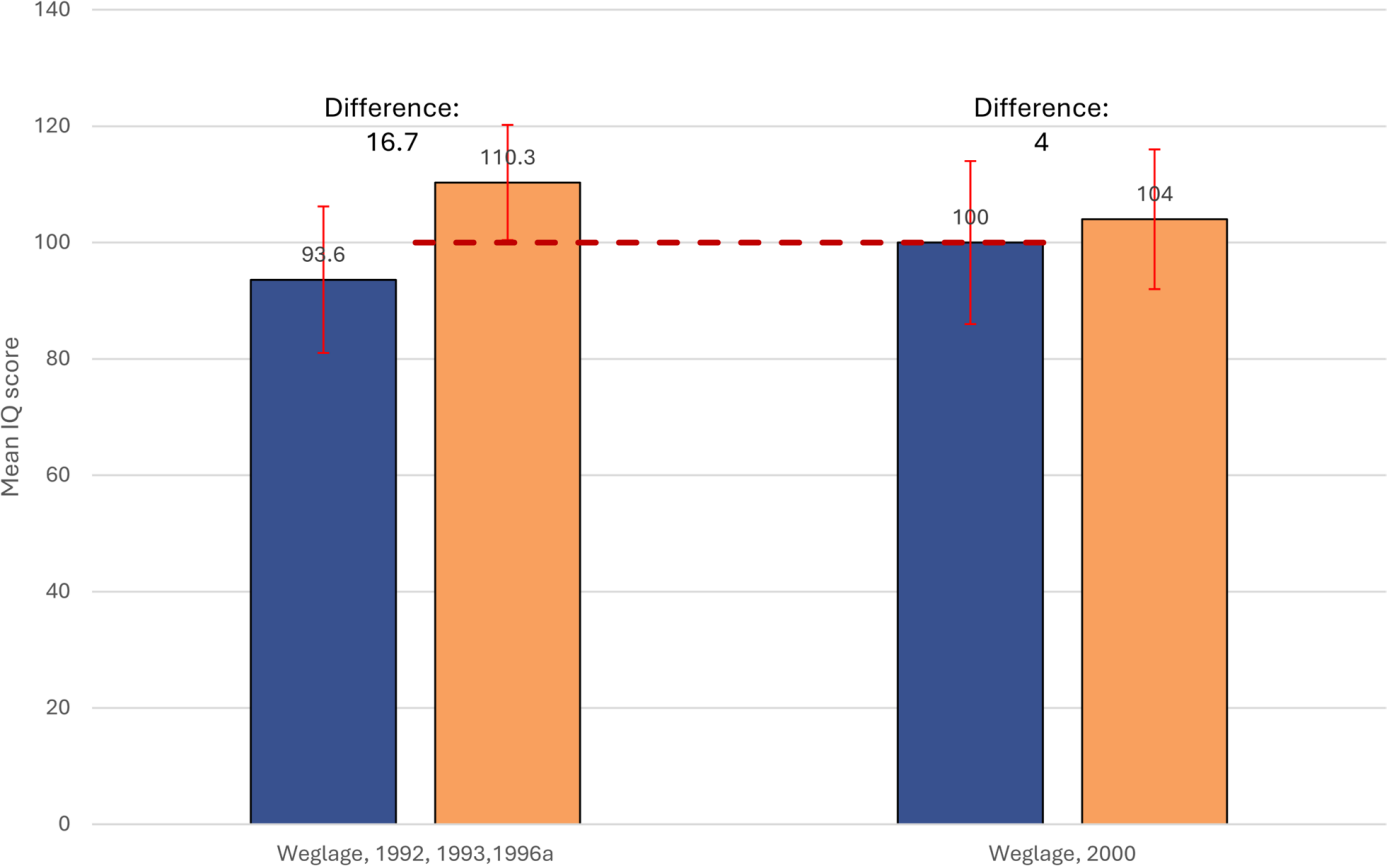
Mean (SD) Culture Fair Intelligence Test scores in restricted subgroups of children with PKU vs external control groups. Dotted line (----) indicates the standardized ‘average intelligence’ score [94]

Two studies by Weglage et al. examined IQ using Culture Fair in children compared to control groups (Fig. 6) [71, 73]. The IQ scores of the control group ranged between 4 and 16.7 points higher than those of PKU patients.

**Fig. 7:**
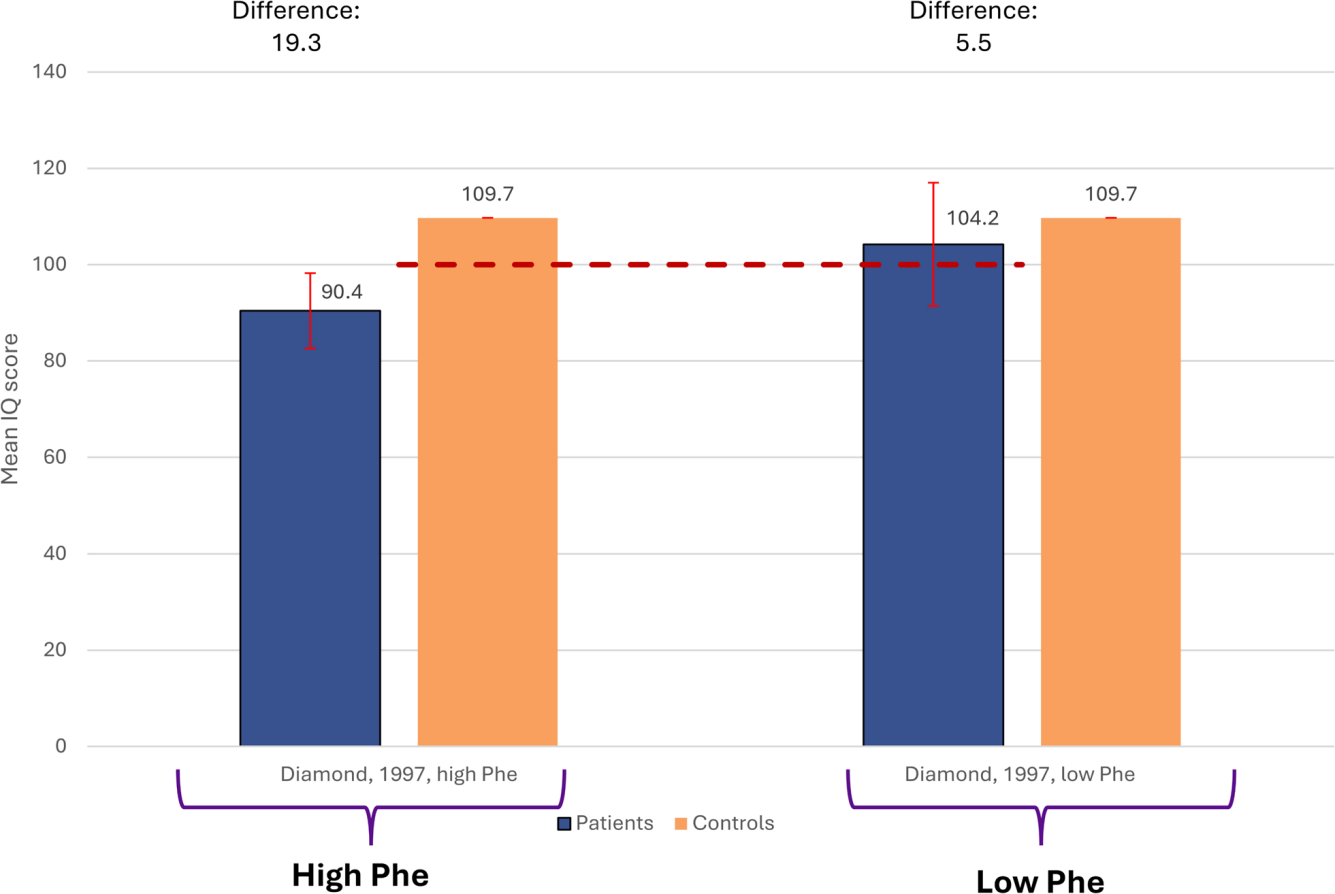
Mean (SD) Stanford Binet Test scores in restricted subgroups of children with PKU vs external control groups. Dotted line (----) indicates standardized ‘average intelligence’ score [95]

Diamond et al*.*, examined the Stanford Binet test against a control group matched on factors including gender, gestational age at birth, birth weight, ethnic background, religion, age at beginning of testing, community of residence, child-care arrangements, number of siblings, birth order, and the age, level of education, and occupational status of each parent (Fig. 7) [70]. Similar to the results described for the other scales, higher scores among the control groups versus PKU patients, including those with high and low Phe, were reported. Higher differences were observed among those with high Phe compared to those with low Phe, which may indicate higher IQ among children with lower Phe.

## Discussion

Effectively managing PKU with traditional early and continuous dietary restrictions to support neurocognitive and psychosocial development is challenging. Despite improvements over time in over time in diet management and a better understanding of the relationship between reducing Phe levels and IQ, discrepancies in cognitive scores between individuals with PKU and the general population persist. Newly approved pharmaceutical treatments such as pegvaliase and sapropterin dihydrochloride, as well as novel emerging treatments such as sepiapterin and gene therapy, may help patients achieve target outcomes and avoid PKU progression [78–80]. Even with available pharmacologic treatments, dietary management is still usually required. Adhering to strict dietary restrictions can be difficult to implement for both patients and their caregivers; consequently, the lack of consistent adherence can affect patient Phe levels and potentially their neurological and cognitive development [14, 15, 81, 82]. The recent ReDAPT study demonstrated that when patients were reintroduced to a diet after a period of no dietary control (mean duration off diet, 19.1 years), time on diet was positively associated with cognitive function using the cognitive proficiency index. However, the regression analysis did not indicate an association with age, time off diet, Phe levels or depression scores with cognitive function.[66] While the sample size of this study was small (n = 9), it does suggest the need for lifetime treatment.

This review revealed that even IQ among those with early treated PKU is affected; almost three quarters of studies reported mean IQ scores below 100 (i.e., below standardized ‘average intelligence’) among individuals with early dietary management for PKU. Considering results from PKU samples in the context of scores from control groups, this review found that scores within overall early PKU samples were 5 to 19.3 points lower compared to control groups when examining Wechsler Intelligence Full scale IQ, and Culture Fair Intelligence Test and Stanford Binet Test comparisons (in children). Furthermore, even when PKU patients reported mean IQ scores of greater than 100, these were still lower than those of controls measured in the same study. These findings are important because lower cognitive function can have a large impact on an individual’s life; within the general population, it has been associated with an increased risk in developing mental health disorders and as a predictor of unsuccessful occupational achievement and prosocial skill deficits [83–85]. Suboptimal IQ, other executive functioning challenges, and attentional concerns that are reported among those with PKU may have an impact on activities of daily living, and result in educational and occupational difficulties [86]. Lower IQ in PKU patients has also been shown to have an impact on health-related quality of life (HRQoL). One recent study reported the overall impact of PKU to be higher among those with lower IQ using the “Phenylketonuria—quality of life” questionnaire (PKU-QoL) [87].

While lower IQ scores were generally observed among those with high Phe levels, the varying subgroups and ages reported hinder the ability to make comparisons. Findings among adults were largely consistent with those reported among children. However, larger differences between PKU and control groups were observed among adults, with the exception of continuously diet-adherent adults. This may be due to the smaller and more heterogenous samples. This finding is somewhat in contrast with those from an interventional study by Huijbregts et al., where it was observed that the effect of high concurrent Phe on the neuropsychological tasks performed was reduced as patients got older[88, 89]. However, in that study, younger patients (aged 7–10 years) were compared to adolescents (aged 11–14 years) and not adults. It is possible with the development of more age-specific measures, the underlying factors driving these differences will become clearer.

In both adults and children, higher mean IQ scores were reported among those with lower Phe levels and those who remained on diet (which acts as a proxy for Phe) compared to those with higher Phe levels, those who were off diet, or those who terminated the diet early. Similarly, Channon et al*.* reported subtle yet frequently, statistically, significant differences in cognitive performance between PKU patients who remained on their diet and who were no longer adhering to dietary restrictions [90]. Furthermore, in a study by Koch et al*.* where early diagnosed PKU patients on and off diet were followed, a lower IQ was reported among those who were no longer adhering to their diet [91]. These findings help demonstrate the close link between cognitive function and blood Phe in PKU.

The findings of this review extend on cognitive results for PKU samples reported previously. In a 2007 meta-analysis of neuropsychological symptoms by Moyle et al., significantly lower full scale IQ scores were observed in PKU patients compared to controls. However, Moyle et al., did not limit their samples to those who were early diagnosed and treated [32]. A more recent meta-analysis by Romani et al., which focused exclusively on early diagnosed adults with PKU, also reported worse performance of PKU groups compared to controls when examining IQ and other cognitive outcomes. However, the review by Romani et al*.* had a broader inclusion criterion than our study, as it did not require Phe levels of > 600 μmol/L at diagnosis. Furthermore, Romani et al. only included studies where patients with PKU and individuals included in control groups were ‘roughly’ matched for IQ, which may bias the results [92].

In alignment with the findings from our review, Waisbren et al., demonstrated that for every 100 μmol/l increase in Phe there was a predicted 1.9- to 4.1-point reduction in IQ [26]. The importance of Phe for IQ is further highlighted in a more recent study by Hood et al., which examined the relationship between cognition and indices of Phe control across the lifetime. They concluded that IQ scores decreased by an average of 5 points for every 100 μmol/L increase in Phe over the lifetime (based on retrospective analysis of medical records for a cohort of children ranging in age from 6 to 18 years), highlighting the importance of good Phe control through dietary management and treatment [33]. Hood et al. also highlighted that performance on IQ was more negatively affected by variability in Phe rather than average Phe, suggesting that avoiding variability in Phe is important [33]. This is consistent with evidence from Anastasoaie et al. that demonstrated that variability in blood Phe levels, rather than average lifetime levels, is more closely related to cognitive outcomes [52].

### Strengths and Limitations

Strengths of this study included the rigorous systematic review methodology employed. While other systematic reviews have included studies of differing designs, the present study focused exclusively on observational studies, the benefits of which include longer follow-up (compared to trials), real-world data insights into treatment adherence and its impact, and more generalizable samples (strict inclusion criteria are not required). This review was restricted to studies that did not include HPA patients and to those on ‘early’ PKU treatment. Additionally, this study included multiple measures of IQ; the findings were strengthened by the similar trends noted across measures, specifically around comparison to control groups. Finally, the synthesis of subgroup analyses across the publications revealed important insights into the changing effects of PKU on cognition across different dietary management approaches.

With regards to limitations, as this SLR relied on the published literature, there is the inherent risk for publication bias. To mitigate this, an extensive search of relevant conferences of interest was conducted to capture studies which may not have been published in a peer reviewed journal article. In addition, as this SLR was restricted to English-language publications, any relevant non-English studies would not have been included. Third, this review included articles that reported on patients who were treated at an early age with dietary or pharmaceutical treatments; patients had to have been classed as “early treated” in the included articles or noted that they were diagnosed and treated within three months of age. For older studies “three months of age” was the standard definition used for early treatment while newer studies use a definition of treatment within 3 weeks [12]. As such, there may be some variability in the definition of early treatment within individual studies. This may have an impact on cognitive ability and IQ level results in articles and therefore have an impact on the results presented here [12]. It is notable however, that even newer studies with cohorts treated from birth are showing early treatment alone is not enough to forestall cognitive impact and other metabolic and environmental factors may play a role [67]. As more long-term data become available on patients who began treatment before 3 weeks, future work should provide more insight on the impact of adherence to treatment and diet management (or lack thereof) on intelligence. Finally, many of the included studies used the Wechsler family of IQ tests. As these are all standardized measures, versions of the Wechsler IQ tests were stratified only into “adult” and “child” IQ tests. However, given the various age-group specific and older/revised versions of these tests, there may be some comparability issues which might impact the results of this study. Although this study attempted to combine data from articles which likely reported on the same population of patients where possible, there may be some overlap of patients between some of the studies due to the small number of patients with PKU and the scarcity of the evidence in this area.

## Conclusions

The present study systematically reviewed data on IQ among early-treated patients with PKU. Findings highlighted that while individual IQ scores may vary, a large proportion of studies reported mean IQ scores from PKU patients of < 100 points. Additionally, mean IQ scores among those with PKU were consistently lower compared to control groups on multiple different IQ scales. Despite that all patients in this review received early treatment, those with poor dietary adherence and higher Phe levels tended to show poorer cognitive ability. Taken together, these data highlight that cognition is affected in PKU, despite early dietary management. Novel pharmacologic treatments are needed that reduce Phe levels such that the burden of neurocognitive impairment in PKU can be alleviated.

## Supplementary Information

Supplementary Material 1

## Data Availability

Datasets generated in this review are available from the corresponding author upon reasonable request.
