# Supplementary material for: Intelligence quotient scores among early-treated phenylketonuria patients: results from a systematic literature review
Source: Orphanet J Rare Dis. 2025 Jun 20;20:314. doi: 10.1186/s13023-025-03830-0 (PMC12181871; doi:10.1186/s13023-025-03830-0)
Supplement: Supplementary file 1 — Supplementary Material 1 [file 13023_2025_3830_MOESM1_ESM.pdf]

Figure S1: Mean (SD) Wechsler Intelligence Full scale IQ in restricted subgroups of children defined by dietary control

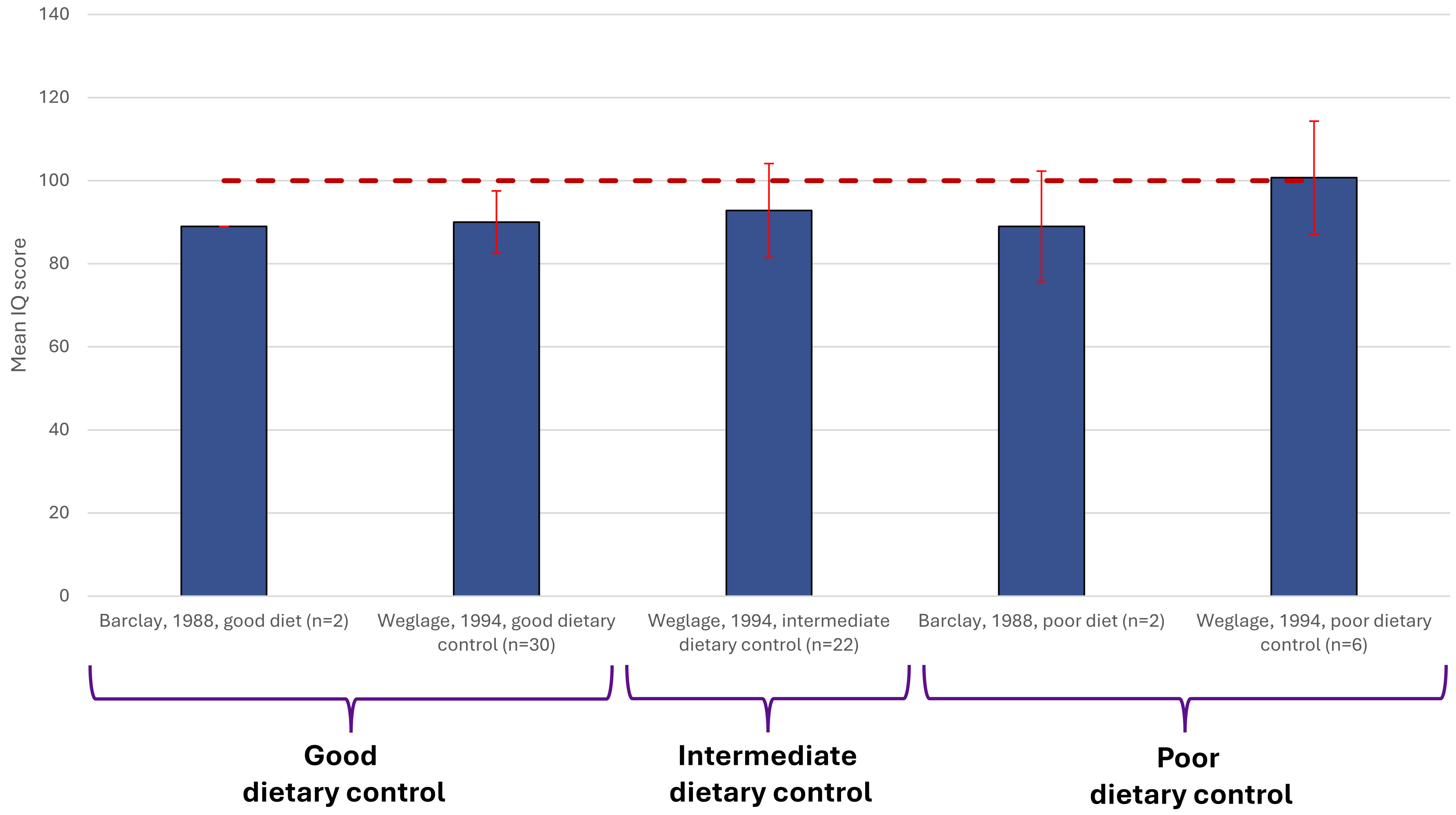

Figure S2: IQ scores among early-treated children with optimal and suboptimal Phe levels

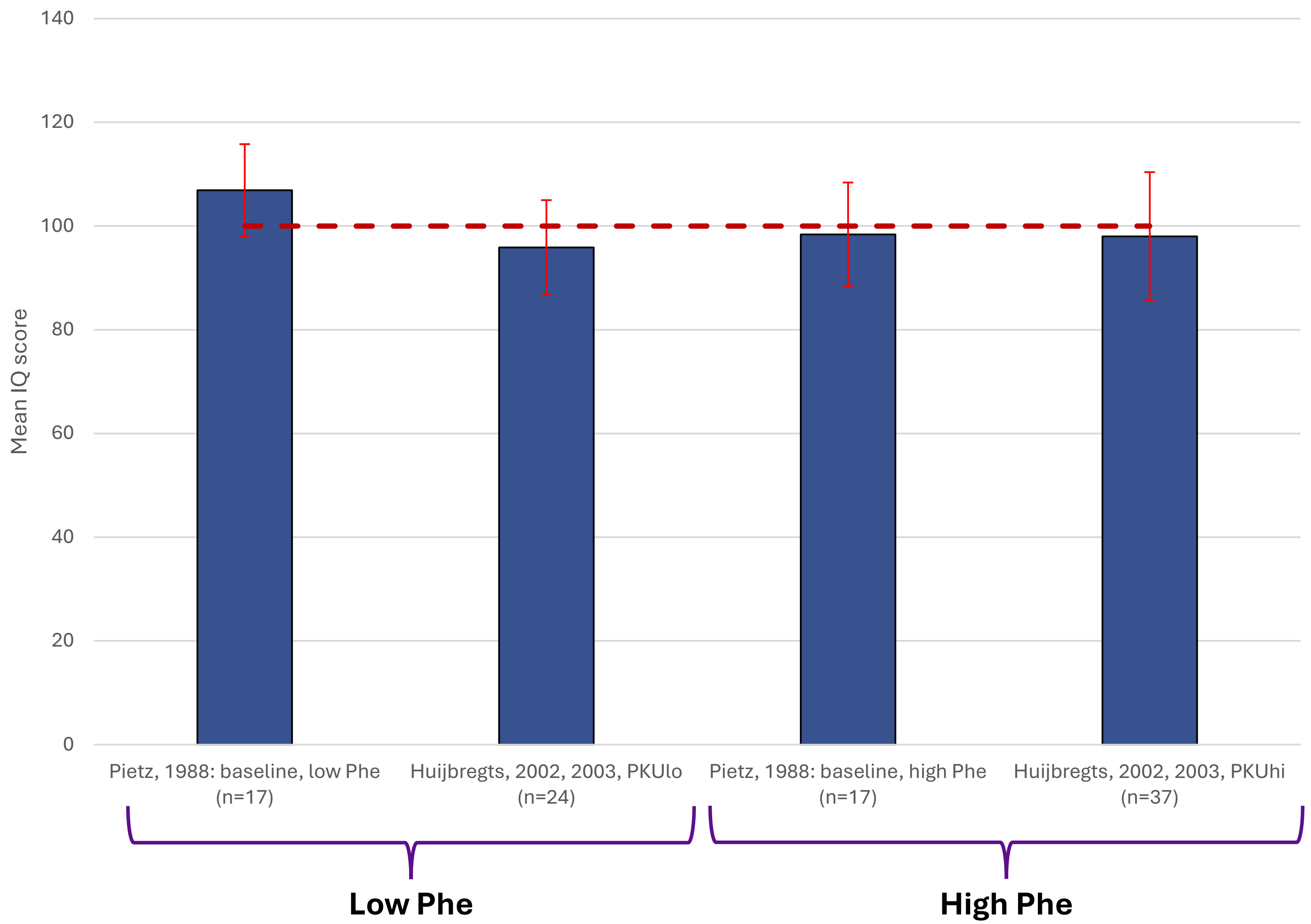

Note: Pietz *et al.* defined low Phe as a mean index of dietry control (IDC) of 4.7 milligrams per deciliter (mg/dl) and high (elevated) Phe as a mean IDC of 9.0mg/dl; Huijbregts *et al.* defined low Phe as ≤360 micromole per liter (µmol/L) and high Phe as >360 µmol/L

Figure S3: IQ among subgroups of children with PKU defined by dietary adherence

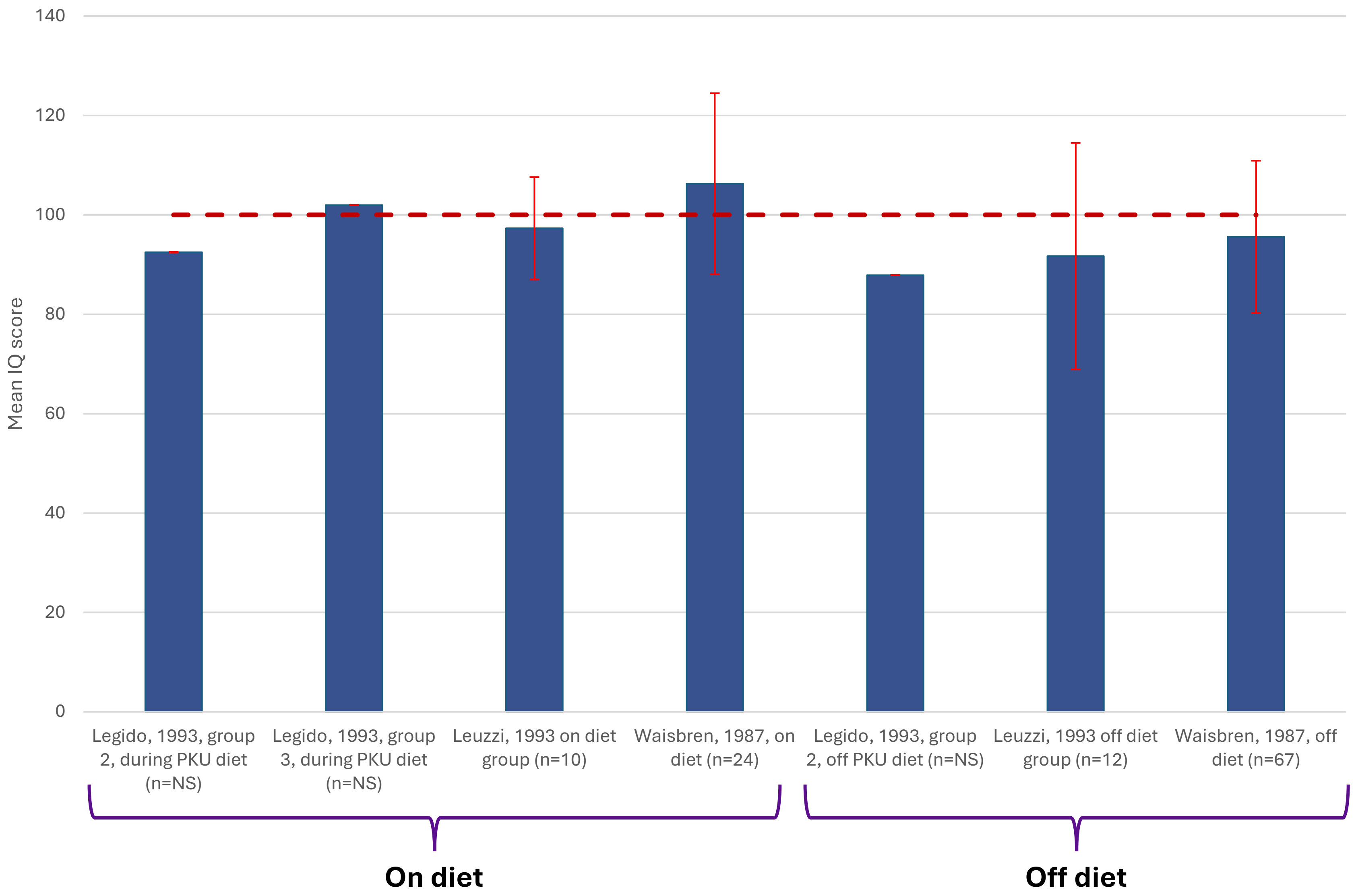

Note: Legido *et al.* group 2 (n=18) initiated a PKU diet treatment before 3 months and had loss of diet control (defined as the age at the beginning of the first six-month period in which the index of dietary control exceeded 15 mg/dL) at a mean of 5 years and group 3 (n=16) initiated diet management before 3 months and had diet control that continues [at time of reporting] at a mean of 11 years; Leuzzi et al. divided their cohort based on the quality of dietary control before and at the moment of of the study using a cut off Phe value of 900 flmoVL with those on or below considered to be on diet and those about to be off diet. On and off diet criteria for Waisbren et al. was not discussed in detail but it was mentioned that 67 individuals discontinued the diet at five years of age and had been off diet for a mean of 7.3 ± 4.1 years at the time of the study, and 24 had remained on the diet.

Figure S4: Mean (SD) Culture Fair Intelligence Test Scale 2 in children

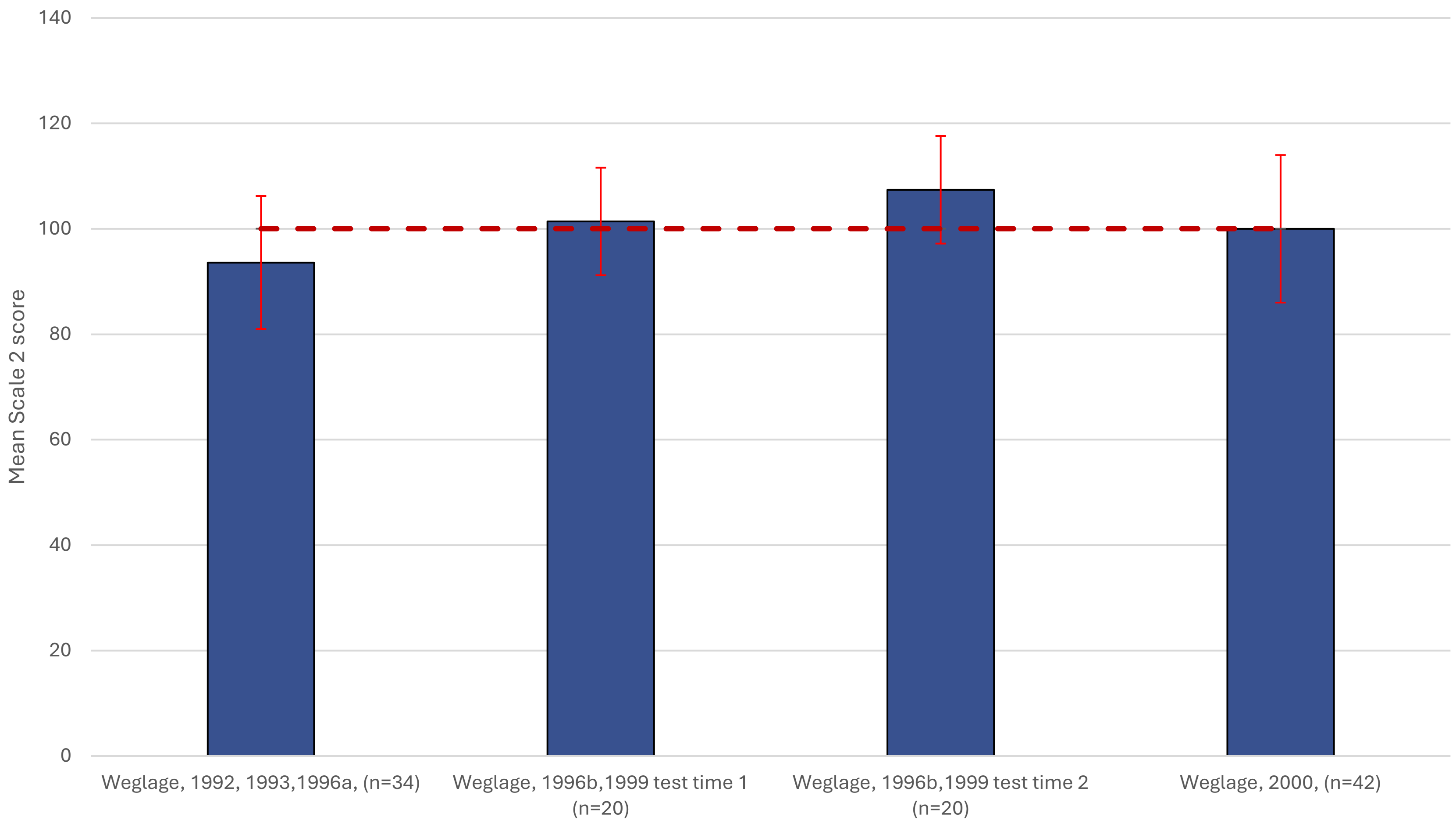

Figure S5: Mean (SD) Wechsler Intelligence Full scale IQ scores in restricted subgroups of adults defined by dietary adherence

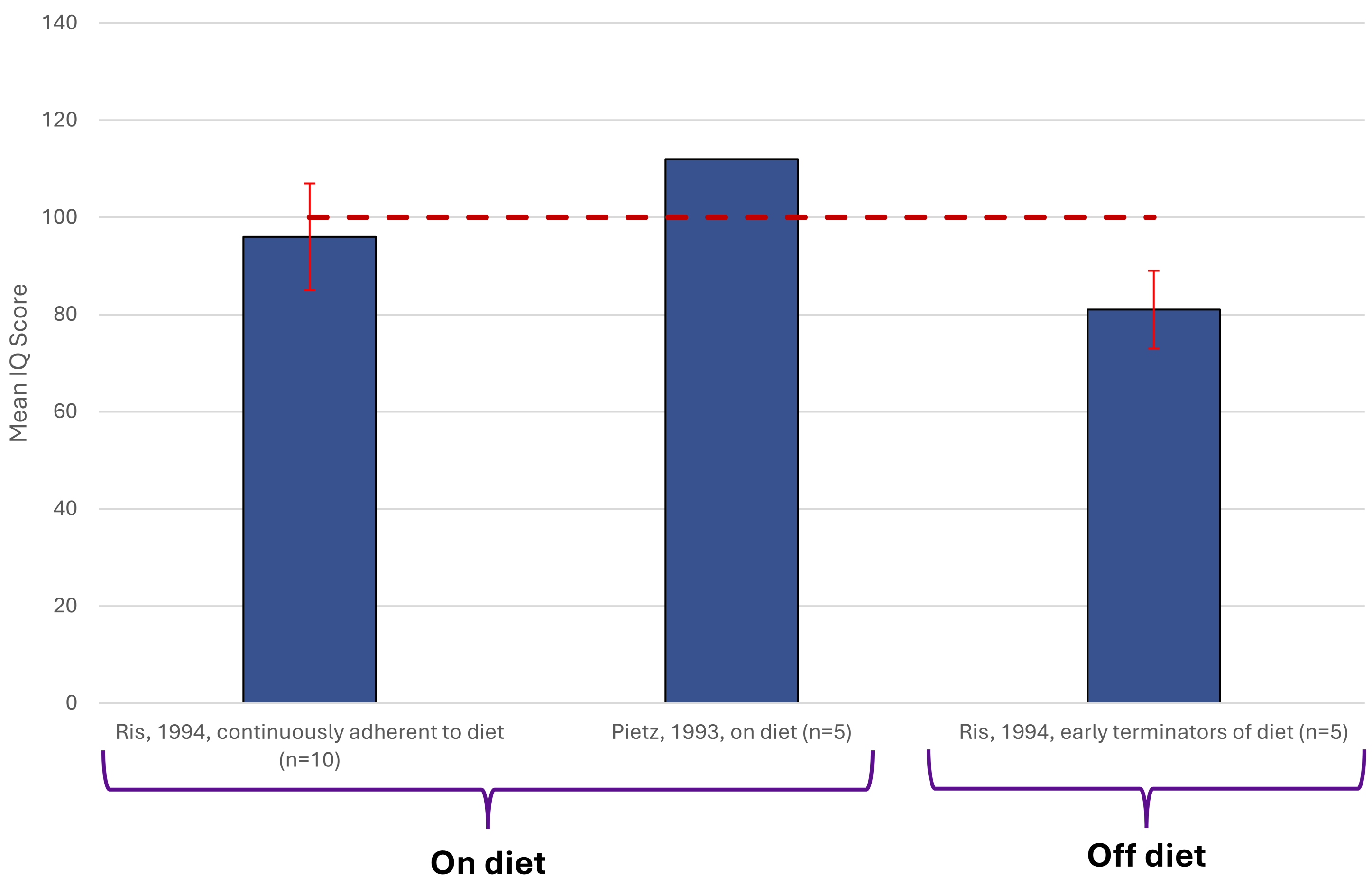

Table S1 Search strategy: Ovid MEDLINE(R) and Epub Ahead of Print, In-Process, In-Data-Review & Other Non-Indexed Citations, Daily and Versions from 1946 to March 01, 2023

1	exp Phenylketonurias/	7241
2	PKU.mp.	3247
3	(Hyperphenylalaninemia or Hyperphenylalaninaemia or Hyper-phenylalaninaemia or Hyperphenylalaninaemias or Hyper-phenylalaninaemias).mp.	1374
4	(phenylketonuria or Phenylketonurias or phenylketonuri*).mp.	8602
5	(Classic Phenylketonuria or classic* Phenylketonuria).mp.	314
6	Classical Phenylketonuria.mp.	230
7	(Phenylalanine Hydroxylase Deficiency or Phenylalanine Hydroxylase Deficiency Disease or Phenylalanine 4-Hydroxylase or Phenylalanine 4 Hydroxylase or PAH deficiency).mp.	342
8	or/1-7	9129
9	exp Epidemiologic studies/ or (epidemiologic$ adj (study or studies)).tw.	3153669
10	exp casecontrol studies/ or (Case control or case-control).tw.	150800
11	exp cohort studies/ or (cohort adj (study or studies)).tw. or Cohort analy$.tw.	2536356
12	((observational adj (study or studies)) or (Follow?up adj (study or studies))).tw.	155423
13	(Retrospective or prospective).tw.	1364726
14	Cross sectional studies/ or (Cross sectional or cross-sectional).tw.	631592
15	longitudinal studies/ or longitudinal.tw.	373602
16	observational study/	138746
17	or/9-16	3956756
18	letter/ or editorial/ or news/ or comment/	2336376
19	(letter or comment*).ti.	185341
20	animals/ not humans/	5064035
21	exp historical article/	409441
22	anecdotes as topic/	4747
23	case study/ or case report/	2321337
24	or/18-23	9812169
25	8 and 17	970
26	25 not 24	912
27	limit 26 to english	818

Table S2 Search strategy from Embase 1974 to 2023 March 01

1	exp Phenylketonurias/	9164
2	exp Hyperphenylalaninemia/	1800
3	PKU.mp.	4974
4	(Hyperphenylalaninemia or Hyperphenylalaninaemia or Hyper-phenylalaninaemia or Hyperphenylalaninaemias or Hyper-phenylalaninaemias).mp.	2321
5	(phenylketonuria or Phenylketonurias or phenylketonuri*).mp.	10064
6	(Classic Phenylketonuria or classic* Phenylketonuria).mp.	395
7	Classical Phenylketonuria.mp.	291
8	(Phenylalanine Hydroxylase Deficiency or Phenylalanine Hydroxylase Deficiency Disease or Phenylalanine 4-Hydroxylase or Phenylalanine 4 Hydroxylase or PAH deficiency).mp.	478
9	or/1-8	11183
10	clinical study/	162198
11	case control study/ or ((case control or case-control) adj (study or studies)).tw.	252242
12	Cohort analysis/ or (cohort adj (study or studies)).mp.	1090525
13	longitudinal study/ or (follow?up adj (study or studies)).tw.	190842
14	retrospective study/	1428994
15	Cross sectional studies/ or ((cross sectional or cross-sectional) adj (study or studies)).tw.	531146
16	Family study/	25753
17	observational study/	318194
18	prospective study/	858608
19	randomized controlled trials/	254630
20	18 not 19	847961
21	(observational adj (study or studies)).tw.	247755
22	(epidemiologic$ adj (study or studies)).tw.	121531
23	or/10-17,20-22	3956596
24	(letter or note or editorial).pt.	2989655
25	(letter or comment*).ti.	238069
26	animal/ not human/	1172005
27	case study/ or case report/	2950370
28	or/24-27	6801489
29	9 and 23	1002
30	29 not 28	942
31	limit 30 to english	895

Table S3 PICOS CRITERIA

**Population**	Individuals with PKU
**Intervention**	Any therapy used in the treatment of PKU, or no therapy
**Outcomes**	• IQ scores, including:<br>• Weschler Intelligence Scale<br>• Culture Fair Intelligence Test<br>• Stanford Binet test
**Study Design**	Retrospective, prospective, or cross-sectional observational studies

Footnote: The population of interest was individuals with PKU. These patients will have defined PKU or Phe levels >600μmol/L at diagnosis. All patients must have received 'early' PKU treatment defined as being within the original article or diagnosed and started treatment within the first 3 months of life.
